# Why Hydrogels Don’t Dribble Water

**DOI:** 10.3390/gels3040043

**Published:** 2017-11-15

**Authors:** Gerald H. Pollack

**Affiliations:** Department of Bioengineering, University of Washington, Seattle, WA 98195, USA; ghp@u.washington.edu; Tel.: +1-206-685-1880

**Keywords:** fourth phase, exclusion zone water, negative charge, protons, swelling, infrared energy, polymer matrix

## Abstract

Hydrogels contain ample amounts of water, with the water-to-solid ratio sometimes reaching tens of thousands of times. How can so much water remain securely lodged within the gel? New findings imply a simple mechanism. Next to hydrophilic surfaces, water transitions into an extensive gel-like phase in which molecules become ordered. This “fourth phase” of water sticks securely to the solid gel matrix, ensuring that the water does not leak out.

## 1. Introduction

Some years ago, faced with the need to define pornography, the US Supreme Court, opined that pornography cannot be defined—but “you know it when you see it”. Similar for gels. We think of hydrogels as containing solid and aqueous phases, but that merely describes the contents of the gel, and not necessarily what a gel really is, or how those two phases interact with one another. Surely you know a gel when you see it, but try crafting a definition.

While I take no stab here at definition, I wish to address some essential features of hydrogels that emerge from the many studies we have carried out and published, and from two relevant books: Cells, Gels, and the Engines of Life [1]; and The Fourth Phase of Water [2]. In these books, I deal extensively with the nature of gel, and particularly the nature of the water lying within (and just outside) the gel. Here, I will deal particularly with solid–water interactions, which determine many of the gel’s principal features.

The interpretations offered here deviate from convention. I will suggest that osmosis has less to do with the gels’ properties than generally thought; and that the newly discovered “fourth phase” of water [2] is central to the gels’ physical features.

## 2. Discovering Water’s Fourth Phase

**Figure 1:**
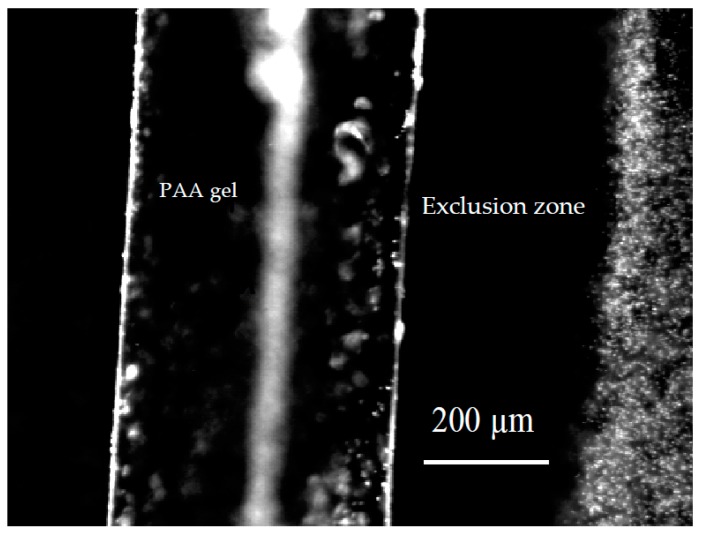
Polyacrylic acid gel immersed in microsphere suspension. Microspheres (right) excluded from a zone, labeled “exclusion zone” or “EZ” next to gel.

In early experiments, we noticed something unexpected. We immersed a polyvinyl alcohol gel into an aqueous microsphere suspension and found that the microspheres were driven from the regions adjacent to the gel. Within about five minutes, a microsphere-free, or exclusion zone (EZ) on the order of 100 µm developed, and persisted for many hours—sometimes even days [3]. Typically, the zone’s width might fluctuate over time, but essentially it remained stable. We then confirmed a similar result with polyacrylic acid gels, which showed even larger exclusion zones (Figure 1).

To our chagrin, we discovered that similar results had been obtained and published in a physiological journal many years earlier [4]. Those investigators studied both natural and artificial lenses of the eye, and noted microsphere-exclusion zones of several hundred micrometers adjacent to them. It soon became clear that exclusion zones were characteristic of many types of gel surfaces and many polymer surfaces, artificial and natural, constituting those gels. It was not just microspheres that were excluded, but also various dyes and other substances [2]. EZs were common features of water next to hydrophilic surfaces.

With earlier evidence of “structured” water in biological systems [5], we began considering whether EZs might represent zones of structured, or ordered, water. Like ice crystals, which exclude particles and solutes to achieve their pure crystallinity, we considered that the exclusion associated with the EZ might signal the presence of an ordered phase of water. We hypothesized that progressive ordering from the gel surface outward would push out the solutes, leaving the zone of exclusion.

That suggestion was eventually confirmed. In published physical chemical studies carried out over a decade, we found that many properties of EZ water differed markedly from those of bulk water. Those studies are summarized in a recent book [2] and include measurements using NMR, microelectrodes, optical birefringence, optical spectroscopy, falling-ball viscometry, infrared imaging, and others. We began thinking of the EZ as a distinct phase of water because it satisfied the requirements for a phase: it was bounded, and it responded to temperature and pressure, as phases must. While the conventional view of water implies solid, liquid, and vapor phases only, this fourth phase appeared to be an ordered, liquid-crystalline phase. It may well correspond to the non-freezing, bound water, long thought to reside next to polymeric surfaces.

**Figure 2:**
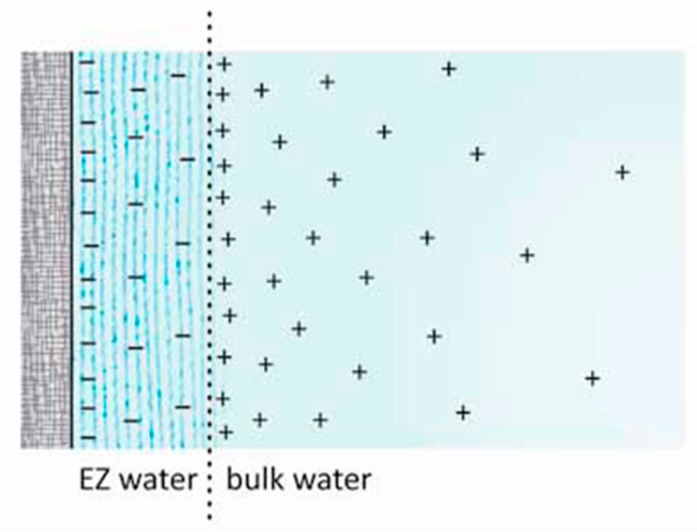
Diagrammatic representation of EZ water, negatively charged, and the positively charged bulk water beyond. Hydrophilic surface at left.

Two principal features characterize this phase (Figure 2). First, the phase is not neutral, as is H_2_O. Commonly, it is negatively charged, with the region of water beyond the EZ containing complementary positive charge [6]. We surmise that the separation occurs as water molecules break into H^+^ and OH^−^, the latter coming together to build the negatively charged exclusion zone, while the protons remain in the bulk water in the form of hydronium ions. Breaking the water molecule into its components has plenty of precedent: it is the initial step in photosynthesis.

The second notable feature is the energy buildup. Creating order and a separating charge both require energy, and we found that the energy comes from light [7]. Spectral measurements showed that all wavelengths explored, from 300 to 5000 nm, contribute, with the most effective lying in the infrared region at approximately 3000 nm (3 µm). Hence, infrared energy is the most efficient energy for building water’s fourth phase. No surprise, because it is the wavelength most absorbed by water.

So, ordered EZ water commonly bears negative charge and is built principally by infrared energy. This energy is freely available in the environment; hence, EZ water should be plentiful.

## 3. Does the Gel’s Interior Contain EZ Water?

Finding gel surfaces lined with EZ water raises questions about the gels’ interior. Is the EZ merely a surface coating, or does EZ water also populate the inside of the gel? Several arguments point to the presence of abundant EZ water inside the gel.

First, consider the nature of the EZ-water template. Since EZs typically grow from hydrophilic surfaces, we surmised that the charges on those surfaces might bear responsibility for nucleating EZ growth. They would act as a template for buildup. We tested the templating idea by determining whether hydrophilic monolayers could nucleate EZ growth. The result was positive [6]. Hence, we surmise that the responsibility for nucleating EZ growth lies with surfaces.

It seems logical, then, that EZ water could build wherever a polymeric surface exists. That would certainly be on the gel’s surface, as observed; but, it should also be inside the gels, where polymeric strands face similar fluid-filled spaces. Little difference should exist between polymers lying on the gel surface and polymers lying inside the gel: both face aqueous zones. Hence, the demonstrated capacity to build EZ water on the exterior implies the same ability in the interior. By this reasoning, the water within the gel should be either partially or fully EZ water.

A second argument for EZ water within the gel comes from measurements of electrical potential. Relative to the exterior, the interior electrical potentials of gels with anionic polymers are typically negative [8,9,10,11,12,13,14,15,16]. While various theoretical arguments can account for this negative electrical potential, a simple argument is that the gel is filled with EZ water, which is negatively charged. If the major component of the gel—water—bears a negative charge, then likely the gel will bear a similar negative charge.

A related issue pertains to cells [17]. The electrical potential of many gels and many cells are in a similar range (negative 50–200 mV). Cells closely resemble gels, the principal difference being the presence of a membrane surrounding the cell but not the gel [1]). It is thought that that membrane bears responsibility for the cell potential. Since the electrical potential magnitudes of cells and gels are similar, however, one might question the widely-held view that the cell potential arises from membrane pumps and channels. If that view were correct for the cell, then what creates the potential of the gel? On the other hand, if both potentials arise from the presence of interior EZ water, then the paradox resolves.

A third consideration arises from the mechanical features of gels. We may think of the gel-like character as arising from the viscoelastic properties of the polymeric matrix. While that view may be logical for gels with limited amounts of water, the argument becomes less rational with gels that are up to 20,000 water to polymer by volume [18]. Could one logically argue that the mere trace of polymer adequately explains the gel’s physical features? Or, is it more likely that the gel’s principal component must create the gel’s characteristic mechanical features?

**Figure 3:**
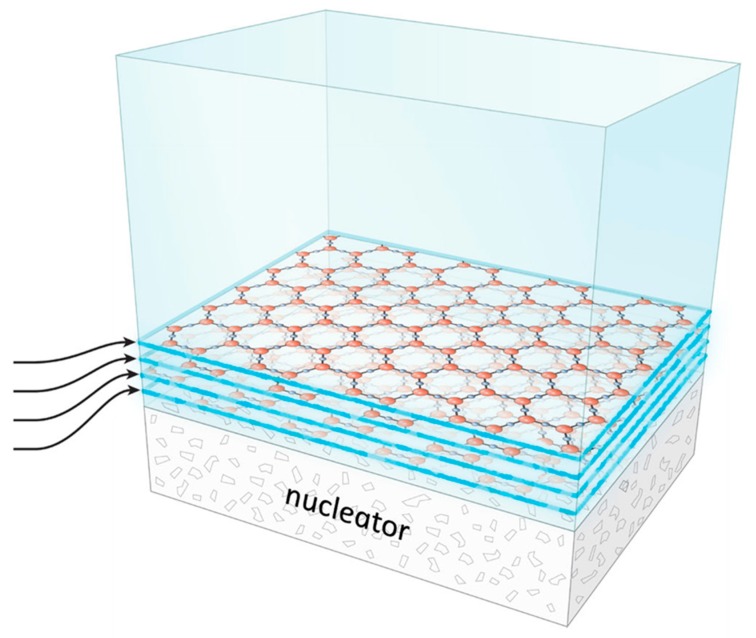
Buildup of honeycomb planes from bulk water (**top**, blue). Hydrophilic surface nucleates EZ growth, which progresses layer by layer.

If it is the water that is responsible, then, the water itself must have a gel-like character. Bulk water has no such character; but EZ water should have exactly that character. That is because of its structure (Figure 3). That structure consists of stacked honeycomb sheets [2]. The hexagons of adjacent honeycomb sheets are offset from one another to juxtapose opposite charges from contiguous sheets. Hence, the sheets stick together, albeit weakly.

This structure gives rise to a characteristic gel-like feature, which can be envisioned in raw egg white as a physical example. A small imposed shear will slightly displace one plane from the next, which will return once the shear force is withdrawn. Larger shear force will break inter-planar bonds, displacing one sheet from another and effectively producing flow. Hence, the EZ structure predicts common gel-like behavior. This feature constitutes a third argument for the presence of EZ water inside the gel.

A fourth argument relates to swelling. Hydrogels take up enormous amounts of water. The usual explanation relates to osmotic drive: polymeric surfaces draw water. The osmotic mechanism may make sense for gels with limited amounts of water; it makes less sense in situations in which the water to polymer ratio is in the order of 20,000 to one [18]. Could a few wispy strands of polymer create an osmotic draw sufficient to attract so huge a volume of water? On the other hand, the EZ explanation seems more plausible: hydrophilic surfaces can demonstrably order and bind millions of molecular layers of EZ water [2]. Hence, the EZ mechanism offers a straightforward explanation for extensive gel swelling. A gel will swell until its cross-links prevent any further expansion.

In sum, four considerations argue for the presence of EZ water inside hydrogels. First, the hydrophilic surfaces of the gel interior are the kind generally responsible for buildup of fourth phase water. Second, fourth phase water is typically negatively charged, and so are most common gels. Third, the mechanical properties of gels match those expected from EZ water. Fourth, the EZ mechanism explains gel swelling, even in gels with huge water-to-solid ratios.

Although these considerations argue for the presence of EZ water inside gels, they do not necessarily argue that all water inside the gel is EZ water. When EZs build, protons get released into the bulk water beyond. Hence, pockets of protonated water may exist within gels. Since protons repel, many protons should be ejected from the gel. Hence, the residual proton concentration is not theoretically set, and may well depend on the type of gel.

## 4. Dribbling Water?

### 4.1. Why Then Should Water Remain within the Gel?

**Figure 4:**
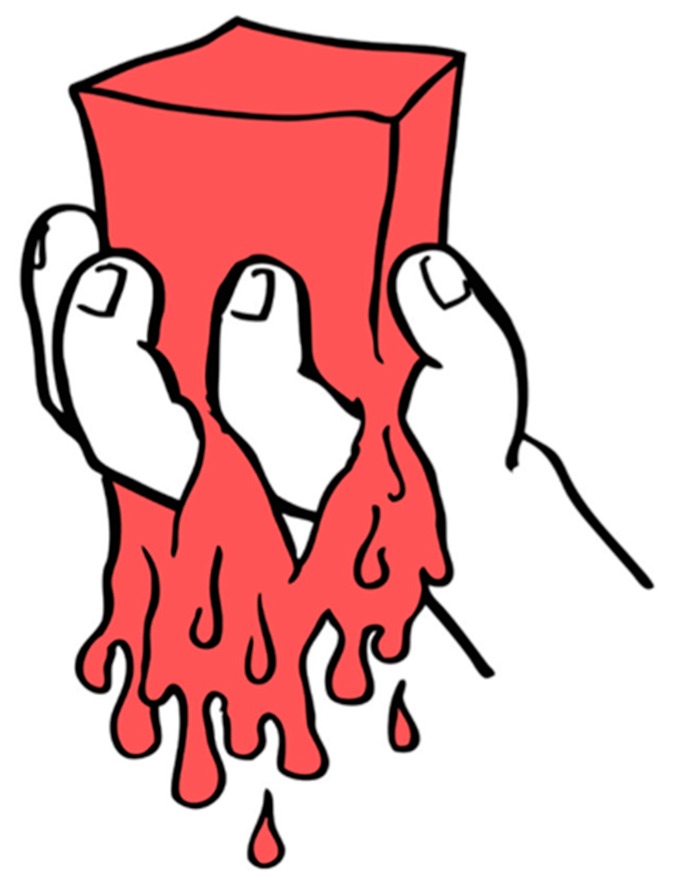
The specter of the leaking gel—averted because EZ water layers stick to polymeric surfaces within the gel.

If the water inside the gel is EZ water, then the answer emerges naturally. EZ sheets stick to their nucleating surfaces, and to one another. Thus, the entire EZ complex is stuck to the polymeric matrix. The matrix should not leak (Figure 4).

### 4.2. Functional Role of EZ Water Inside Gels?

**Figure 5:**
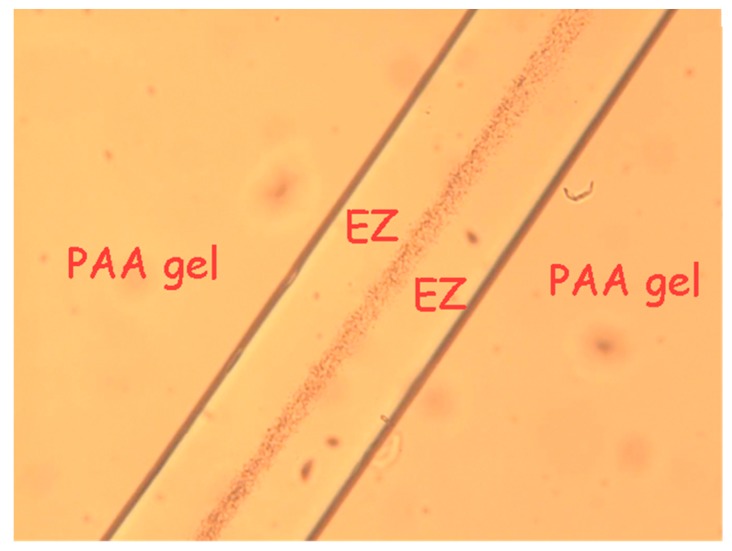
Flow in tunnel bored within polyacrylic-acid gel. EZ forms adjacent to gel material, while aqueous microsphere suspension resides in core. Microsphere suspension flows.

In our laboratory, we have been able to visualize EZ water inside gels under certain conditions [19,20]. In one example, polyacrylic acid gels were formed with a narrow wire inserted prior to gelation. As the material gelled, we pulled the wires, creating tunnels inside the gels. When the gels were immersed in an aqueous microsphere suspension, an annular EZ could be visualized adjacent to the gel surface. Figure 5 shows an example.

In this figure, note that the microspheres are excluded from the annular region adjacent to the gel; they are confined to the narrow channel at the center. The microspheres serve as markers for flow visualization. Videos show a steady flow of microspheres and water along that interior channel—the so-called “self-driven” flow. Light drives that flow; increasing light speeds it by up to five times [20]. We found much the same flow with hydrophilic tubes made of Nafion [19]; the flow runs continuously through the tube, often without stopping for more than a full day. More recently, we confirmed the flow inside tunnels formed from a series of different hydrogels [21]. Hence, the light-driven-flow phenomenon is general; and, it is driven by light.

Mechanistically, we found that the flow resulted from the protons generated as consequence of EZ growth. Those protons lie in the tunnel’s central core (denoted by the presence of microspheres). Repulsion among protons creates a pressure, which pushes the protons out of one end of the tube or the other. Once that flow begins, additional water gets drawn in from the opposite end of the tube, perpetuating the process. Many applications of this flow principle can be envisioned, ranging from drainage, to propulsion, even to the driving of blood through vessels.

Hence, the presence of EZ water inside gels is not only fundamental for understanding the gels’ physical properties, but also for providing a basis for practical functions of all kind. It also prevents leakage from the gel.
